# Impact of multiplicity of infection and baculovirus co-infection ratio on recombinant adeno-associated virus vector production in insect cells

**DOI:** 10.3389/fbioe.2025.1679358

**Published:** 2025-11-21

**Authors:** Mels Schrama, Linda A. de Jong, Stefan Kint, Pranav Puri, Femke Hoeksema, Gorben P. Pijlman, Monique M. van Oers, Rene H. Wijffels, Dirk E. Martens

**Affiliations:** 1 Bioprocess Engineering, Wageningen University, Wageningen, Netherlands; 2 Laboratory of Virology, Wageningen University, Wageningen, Netherlands; 3 VectorY Therapeutics B.V., Amsterdam, Netherlands

**Keywords:** recombinant AAV, baculovirus expression vector system, co-infection, gene therapy, multiplicity of infection

## Abstract

Recombinant adeno-associated virus vectors (rAAVs) play an important role in gene therapy for the effective delivery of therapeutic genes into target cells. The Baculovirus Expression Vector System (BEVS) has gained significant attention for its versatility and scalability as an rAAV production platform. The existing Dual-Bac system uses two separate baculovirus constructs (Bac-GOI-ITR and Bac-Rep-Cap), each carrying essential genetic elements for rAAV production in insect cells. This study investigated how two infection parameters of the Dual-Bac system, the total Multiplicity of Infection (MOI) and the baculovirus co-infection ratio, influence rAAV production efficiency. Different budded virus (BV) concentrations were used to explore the effects on assembled rAAV capsid and encapsidated transgenic genome yields. An excess of Bac-Rep-Cap in synchronous co-infection produced high-quality rAAVs, whereas increasing the ratio of Bac-GOI-ITR to Bac-Rep-Cap resulted in more empty rAAV capsids. The optimal BV ratio varied with the total MOI used for co-infection, and when applying a BV ratio of one, MOI variations had a minimal impact on rAAV quality. These findings highlight the importance of optimizing the MOI and BV ratio to enhance rAAV yield and quality, contributing to more robust gene therapy production.

## Introduction

Recombinant adeno-associated virus vectors (rAAVs) have several beneficial characteristics regarding their *in vivo* application; a range of different cell types can be targeted by using the existing AAV serotypes, AAVs have low immunogenicity, are non-pathogenic, and recombinant AAV genomes remain primarily episomal upon transduction while still inducing stable expression of the gene of interest (Wu et al., 2006).

The baculovirus expression vector system (BEVS) has emerged as a versatile platform for producing rAAVs in large quantities, serving as an attractive alternative to mammalian expression systems (Joshi et al., 2021; Liu et al., 2024; Urabe et al., 2002; Ylä-Herttuala, 2012). The BEVS platform typically uses *Spodoptera frugiperda 9* (Sf9) in combination with a genetically modified baculovirus, usually *Autographa californica* multiple nucleopolyhedrovirus (AcMNPV). This recombinant AcMNPV has a large double-stranded DNA genome (∼134 kilobases), which allows for transgenic sequences of substantial size to be expressed (Ayres et al., 1994; Van Oers, 2006). To produce recombinant proteins or AAV vectors, a baculovirus is constructed that contains the transgene under the control of the strong baculovirus polyhedrin or p10 promoter (Van Oers et al., 2015). The recombinant baculovirus replicates in the nucleus of the insect cells, and budded viruses (BVs) are produced that spread the infection to neighboring cells.

The production of rAAV requires at least three essential elements to be present in the producer cell: AAV replication proteins (Rep52 and 78), capsid proteins (Cap = VP1, 2, and 3), and a therapeutic transgene cassette (with a Gene of interest = GOI) flanked by AAV inverted terminal repeats (ITRs). Rep52 plays a role in AAV genome packaging, and Rep78 in AAV genome replication (King et al., 2001; Smith et al., 1997). The Cap proteins together form the icosahedral rAAV capsid particle that encapsidates the recombinant genome. The single-stranded rAAV genome includes a transgene cassette that carries the desired genetic material, controlled by a suitable promoter located between the ITRs. All these elements (Rep, Cap, and GOI) are needed at different levels and times during the infection cycle to obtain fully packaged rAAV particles. An imbalance in the rAAV elements can lead to lower rAAV yields or the production of empty capsids (Aucoin et al., 2006).

The first reported use of BEVS for producing rAAVs involved a co-infection strategy with three different baculovirus constructs (Triple-Bac): one baculovirus for the rep gene, another for the cap gene, and a third containing the ITR-flanked transgene cassette (Urabe et al., 2002). The Triple-Bac system was later improved to Dual-Bac (*rep* and *cap* genes in one baculovirus and the transgene in another) and Single-Bac systems (all genes in one baculovirus) (Galibert et al., 2021; Smith et al., 2009).

**FIGURE 1:**
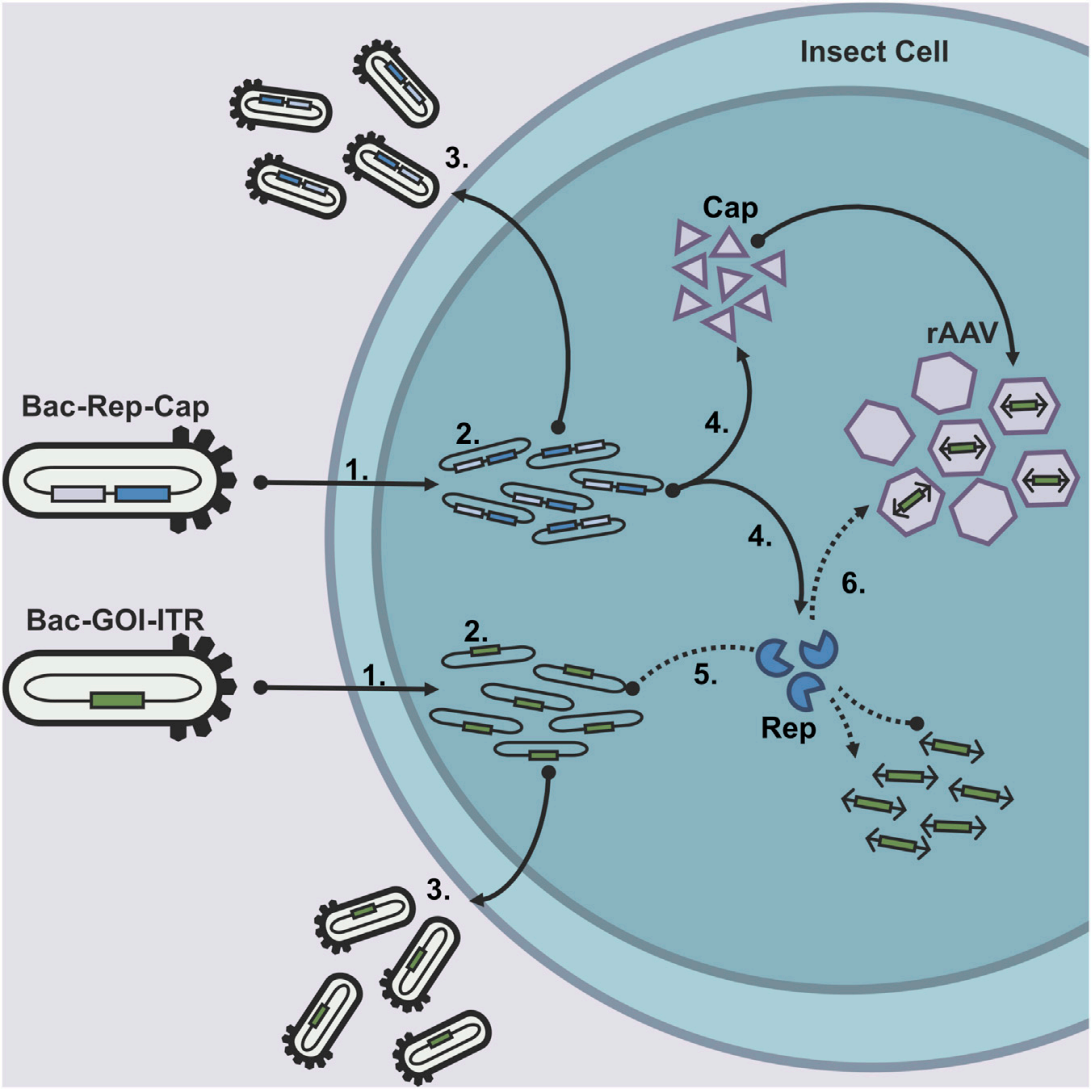
After the recombinant baculovirus constructs have infected an insect cell (1), the Baculovirus genomes replicate (2), followed by subsequent budded virus (BV) production (3). The baculovirus genomes also encode the essential elements for AAV production (Rep, Cap, and ITR-GOI-ITR). Rep and Cap are transcribed and translated at the late stage of the baculovirus infection (4). The rAAV genome DNA is produced from the baculovirus genome by the Rep78 protein (5) and packaged in the rAAV capsids (6) by the Rep52 protein.

When using the Dual-Bac approach for rAAV production, a co-infection is required with the baculovirus harboring the transgene cassette (Bac-GOI-ITR) and the baculovirus harboring the *rep* and *cap* genes (Bac-Rep-Cap) (Figure 1). This setup is commonly used in industrial environments because it simplifies infection and allows platform reuse, where only the transgene baculovirus is replaced with new vector variants (Destro et al., 2023).

After entering the host insect cell, both baculovirus genomes will be replicated before BV release, and late-stage expression of the *rep* and *cap* genes is initiated (Destro et al., 2023). However, the production of the rAAV genome relies on the interplay between the two baculovirus constructs. The replication of single-stranded rAAV DNA-genome copies from Bac-GOI-ITR is done by the Rep78 proteins expressed from Bac-Rep-Cap. The concurrent production of rAAV genomes, AAV capsid proteins, and Rep52, needed for AAV genome packaging, is required to obtain full, functional rAAV vectors for gene therapeutic purposes.

The multiplicity of infection (MOI; number of infectious viruses per cell) is an essential parameter for controlling baculovirus infection in cell culture processes. It has been shown that by changing the individual MOIs in co-infections, recombinant protein levels can be altered, which can affect the end product quantity and quality (Hu and Bentley, 2001; Palomares et al., 2002). The effect of the ratio between co-infecting baculovirus constructs on rAAV yields has been highlighted in several studies, but that research mainly concerned the Triple-Bac system (Joshi et al., 2021). These reports indicated that variation in the amount of Bac-GOI-ITR minimally affected rAAV yields as opposed to the amount of Bac-Rep and Bac-Cap present, which affected rAAV genome and rAAV particle (capsid) titers, as well as the number of functional rAAV particles (Aucoin et al., 2006).

The probability of infection of an individual cell is described by a Poisson distribution, which assumes each infection event to be independent with no effect on secondary infection events (Hu and Bentley, 2001). When using high MOI strategies (MOI≥5; >99% of cells are infected), synchronous infections are established, meaning all cells become infected simultaneously. However, high MOI strategies require large volumes of BV inoculum and sometimes additional virus concentration steps, increasing the complexity and costs of the production process. This can be solved using a low MOI strategy (MOI<<1), where a small fraction of cells is initially infected. These infected cells release large numbers of progeny BVs that will infect the rest of the cell culture. Low MOI strategies are attractive for large-scale manufacturing because they reduce baculovirus seed needs and simplify upstream logistics related to aliquot size and the lifespan of the virus seed bank (Maranga et al., 2003).

For co-infections, some studies indicate that the infection process is more complex, and the infected cell percentage may not directly be inferred using the Poisson distribution as for single infections (Mena et al., 2007). A phenomenon that can be observed in this situation is the superinfection exclusion effect (Beperet et al., 2014). Superinfection exclusion refers to the restriction of subsequent infections by a different virus construct already present within an infected cell. As superinfection exclusion is a time-dependent effect, it can especially affect (asynchronous) low MOI co-infections where at least one virus construct requires an additional viral replication cycle for full cell culture infection (Beperet et al., 2014; Virgolini et al., 2023). This makes BV release kinetics an important factor to consider for a low MOI co-infection strategy, as potential differences in the release kinetics of each baculovirus construct might be an additional source of variation.

Reports in literature on using low MOI for co-infections with BEVS are scarce, and the outcomes are divergent. A literature report on rAAV production using a Triple-Bac approach showed equivalent volumetric yields for both low and high MOI strategies in the production setup (Mena et al., 2010). However, other articles report decreased yields using a low MOI strategy (Meghrous et al., 2005; Negrete et al., 2007).

Therefore, it remains unclear what the optimal BV ratio is for rAAV production using the Dual-Bac system and whether low MOI conditions are robust enough to be applicable. The objective of the current study is to investigate the effect of the ratio of a Bac-GOI-ITR virus to a Bac-Rep-Cap virus on rAAV production at both high and low MOI conditions. To achieve this, baculoviral replication kinetics, rAAV yields, and expression levels of Rep, Cap, and GOI are studied at different MOI conditions and BV ratios.

## Materials and methods

### Insect cell culture and cell count

Sf9 cells were grown in chemically defined EX-CELL® Insect Cell Medium (Sigma-Aldrich). The insect cells were maintained in 125 mL polycarbonate shake flasks (VWR) with a working volume of 40 mL in a non-humidified incubator at 28 °C on an orbital shaker platform set at 130 rpm (25 mm orbital diameter). The same cultivation settings, volumes, and vessels were applied for all experimental conditions described. A maintenance culture was subcultured twice weekly to keep the cells in the exponential growth phase. The cell density, viability, and cell size were assessed using the Countess™ 3 FL automated cell counter (ThermoFisher Scientific) in combination with trypan blue staining.

### Baculovirus plaque purification, amplification, and titration

Two distinct recombinant baculovirus constructs were used to produce rAAVs. The first baculovirus, named Bac-GFP-ITR, contains the rAAV vector genome consisting of the AAV inverted terminal repeats flanking the gene for enhanced green fluorescent protein (eGFP) as GOI, regulated by the cytomegalovirus (CMV) promoter plus enhancer sequence introduced into the baculoviral ODV-e56 locus (BACe56) by transposition (Pijlman et al., 2020). The second construct was Bac-Rep-Cap as described by Chen (2008).

Both baculovirus constructs were plaque-purified before generating a working stock of clonal viral populations. The virus from individual plaques was amplified by infecting a monolayer of Sf9 cells in six-well plates. Amplified plaques were examined for GFP expression using fluorescence microscopy and for the expression of Rep52, Rep78, and VP1, VP2, and VP3 through Western blot.

Plaque amplifications were further expanded in Sf9 cells grown in 250 mL working volume in 1 L shake flasks (VWR). Cells growing in the exponential phase at a cell density of 2 × 10^6^ cells/mL and viability higher than 90% were infected with one of the baculovirus constructs at an MOI of 0.1 BV per cell. Infected cells were incubated at 28 °C on an orbital shaker platform rotating at 130 rpm. Three days post-infection, cells were removed from the culture by centrifugation at 300 ×g for 5 min, and the supernatant was filtered using a 0.45-μm (pore size) PES filter. The filtrate containing the BVs was supplemented with 10% freezing solution (50% sucrose (Sigma-Aldrich) and 0.01% Pluronic F68 (Gibco)) and stored as 5 mL aliquots at −80 °C for long-term use.

Infectious BV titers (BV/mL) were determined by flow cytometry using a monoclonal antibody targeting the BV envelope protein GP64 (eBioscience™, ThermoFisher Scientific) on infected insect cells as described in the ExpiSf™ Expression System User Guide (ThermoFisher Scientific, Appendix D). In short, a 24-well plate was prepared with a 10-fold dilution range of the test virus stock, after which cells were seeded into the virus-containing wells (reverse infection). After 14–16 h post-infection (hpi), virus-infected cell percentages were determined by flow cytometry based on GP64 detection.

### Release of BVs from Sf9 cells

The BV release kinetics for both baculovirus constructs were determined by measuring BV titers over time. Triplicate shake flask cultures of Sf9 cells at a cell density of 1 × 10^6^ cells/mL were infected with an MOI of 10 and incubated at 28 °C for 1 h followed by a complete medium replacement by centrifugation for 5 min at 300 × g. At specific time points post-infection, up to 48 h, culture fluid samples were harvested (300 × g for 5 min), and the supernatant was used for BV titration by flow cytometry, as previously described, to generate virus release curves.

### Effect of total MOI on rAAV production

The effect of the combined MOI of the Bac-GFP-ITR and Bac-Rep-Cap viruses, further indicated as total MOI, on the production of rAAV was evaluated by co-infecting Sf9 cells at different total MOIs with a constant virus ratio of 1:1. Triplicate shake flask cultures of Sf9 cells at a cell density of 1 × 10^6^ cells/mL were infected with a 100-fold serial dilution of a mixture of equal concentrations of Bac-GFP-ITR and Bac-Rep-Cap. The total MOIs evaluated were 20, 0.2, and 0.002 BV/cell. A cell density of 1 × 10^6^ cells/mL was used at infection to allow 1 day of pre-infection growth from 5 × 10^5^ cells/mL and help prevent nutrient limitation during the production phase. The infected cell cultures were maintained under standard culture conditions for up to 96 hpi. Cell suspension samples (1 mL) were taken daily for cell counting, rAAV characterization, and flow cytometry. At 96 hpi, the remaining culture volume was harvested for rAAV-based affinity purification. An uninfected negative control condition was used to visualize the cell growth curve in the absence of a baculovirus infection. An additional single infected Bac-Rep-Cap condition (MOI 0.001) served as a negative control for genome packaging as this condition does not contain the sequence for the rAAV genome.

### Effect of BV ratio on rAAV production

The impact of the BV ratio on rAAV production was investigated by varying the relative proportions of Bac-GFP-ITR and Bac-Rep-Cap while maintaining a constant total MOI. Keeping the total MOI fixed prevented effects from the infection load itself. This ensured that any variations in rAAV productivity could be ascribed to the relative abundance of Bac-GFP-ITR and Bac-Rep-Cap, rather than the total virus input. This was done for two MOI conditions: a high MOI of 25 for synchronous co-infection and a low MOI of 0.2 for asynchronous co-infection. Triplicate shake flask cultures of Sf9 cells were co-infected with a BV mixture containing variable titer ratios of Bac-GFP-ITR and Bac-Rep-Cap. The specific ratios of the BVs of the two baculovirus constructs were adjusted to 1:9, 1:4, 1:1, 4:1, and 9:1.

For synchronous infection conditions, a minimal individual MOI of 2.5 was selected as it requires a minimal amount of BV stock to be added per culture while still resulting in 92% infection. This minimal individual MOI of 2.5 gives a total MOI of 25 for the ratios tested (MOIs tested: 2.5:22.5, 5:20, 12.5:12.5, 20:5, 22.5:2.5).

For asynchronous infection conditions, a total MOI of 0.2 was selected, as it reduces virus stock usage by more than 100-fold compared to synchronous conditions (MOIs tested: 0.02:0.18, 0.04:0.16, 0.1:0.1, 0.16:0.04, 0.18:0.02).

Triplicate shake flask cultures of Sf9 cells at a cell concentration at infection (CCI) of 1 × 10^6^ cells/mL were infected with the different ratios of BV. The infected cultures were maintained under standard conditions for up to 96 hpi, and daily samples were collected for cell counting, rAAV characterization, and flow cytometry. Based on the observations from the total MOI experiment, it was decided to harvest the culture at 96 hpi for the MOI 0.2 conditions and 72 hpi for the MOI 25 conditions.

### Harvesting and affinity purification

The Sf9 cell suspension was lysed to release the intracellular rAAVs by adding 2% lysis buffer (250 mM MgCl_2_, 0.05% Pluronic F68, and 1% Triton X-100 in 2 M Tris buffer (pH 8)) and Benzonase nuclease (10 U/mL) followed by a 1-h incubation step at 28 °C and 130 rpm before centrifugating the lysate at 3,000 × g for 30 min. The supernatant was stored at −80 °C before affinity purification, qPCR, or ELISA.

For affinity chromatography, triplicate samples were pooled, and the crude lysate was filtered with a 0.45 um filter before loading the column. Purification was performed using an ӒKTA pure™ chromatography system (Cytiva) with a 1 mL POROS™ GoPure™ AAVX Pre-packed Column (ThermoFisher Scientific). Before use, the column was equilibrated with 20 column volumes (CVs) of equilibration buffer (20 mM Tris, 0.5 M NaCl, 0.001% Pluronic F-68, pH 8.0). The crude material was loaded in a sequential application using a 10 mL Superloop (GE Healthcare) at a flow rate of 1 mL/min. This flow rate was maintained throughout the chromatographic procedure. The column was washed with equilibration buffer for 20 CVs. Bound rAAV was eluted from the column by applying an elution buffer (100 mM citric acid, 0.001% Pluronic acid, pH 2.4). Elution fractions (1 mL) were collected into tubes containing 100 µL of 1 M Tris-HCl (pH 9.0) to neutralize the low-pH elution buffer. The first three fractions, containing the purified rAAV, were pooled and stored at −80 °C for further analysis.

### AAV quantification by ELISA

The total amount of rAAV capsids was determined using cell lysate and a commercially available ELISA kit (PRAAV5, Progen). This kit is based on a sandwich ELISA technique. It uses a monoclonal antibody (mAb, ADK5a) specific for a conformational epitope on assembled rAAVs, which is coated onto the plate to capture rAAV particles from the lysate. A biotin-conjugated mAb binds to the captured rAAV particles on the plate, followed by a reaction of streptavidin peroxidase conjugate with the biotin molecules. Adding a specific substrate leads to a color reaction proportional to the amount of bound viral particles.

### Real-time polymerase chain reaction for rAAV genome quantification

Nuclease-resistant rAAV genome titers were determined by quantitative, real-time PCR analysis of crude cell lysates with a commercially available kit (qPCR AAV Titer Kit, Applied Biological Materials Inc., Cat. No. G931, ABM Good) that uses a 3′ITR-specific oligonucleotide pair. The kit provides an assay that employs a DNase step to degrade non-encapsidated DNA, followed by a quick capsid disintegration step before qPCR-based genome detection.

### rAAV full/empty ratio determination with stunner

Purified rAAV samples were concentrated ∼30-fold using a Spin-X UF concentrator (100.000 MWCO, PES, 500 uL, Corning) and analyzed using a Stunner device, using the Gene Therapy application software. The Stunner instrument acquired UV/Visible, dynamic light scattering (DLS), and static light scattering (SLS) data from sample wells. The software parameters encompassed theoretical molar masses for the rAAV capsid and the target packaged DNA needed to determine the percentage of rAAV capsids containing a genome. Technical quadruplicates of the pooled samples were conducted.

### Flow cytometry

Flow cytometry was used to determine the percentage and intensity of GFP-expressing Sf9 cells during rAAV production. Unstained cell culture was analyzed using a CytoFlex flow cytometer (Beckman Coulter) equipped with a 488 nm laser as an excitation source. The green fluorescence emission was detected using a 525/40 nm band-pass filter. At least 10,000 cells gated on forward and side scattering were analyzed per sample. Doublet exclusion was performed by plotting the height against the area for the forward scatter signal. Uninfected cells were used as an auto-fluorescence control to set up the gating parameters. Flow cytometric data was analyzed using the CytExpert Software, and mean fluorescence Intensity (MFI) for all samples was normalized to the MOI10:10 condition at 96 hpi for comparison.

Flow cytometry was also used to detect total AAV Rep expression. Infected Sf9 cells were fixed with 3% paraformaldehyde in PBS for 10 min and subsequently washed three times with ice-cold PBS. Following fixation, cells underwent permeabilization with 0.2% Triton X-100 for 5 min, followed by three washes of 5 min each in PBS. Fixed cells were stained overnight at 4 °C with anti-AAV2 Replicase mouse IgG1 303.9 (1:10, Progen) before staining with Rat anti-Mouse IgG1 Secondary Antibody PE-Cyanine7 (1:200, ThermoFisher: 25-4015-82) for 1 h at room temperature and analyzed using a CytoFlex flow cytometer (Beckman Coulter) with the 780/60 nm bandpass filter. Flow cytometric data were analyzed using the CytExpert Software, and MFI of the stained cell population was normalized to the MOI 12.5:12.5 condition at 48 hpi.

### Statistical analysis

AAV titer data was analyzed using one-way analysis of variance (ANOVA), followed by Tukey’s multiple comparison test to identify significant differences between groups. Before analysis, titer values were log_10_-transformed. Significant differences (p < 0.05) are indicated in figures by letter codes (a, b, c, etc.), with groups sharing the same letter not being significantly different.

## Results

### Bac-Rep-Cap and bac-GFP-ITR BV release kinetics

**FIGURE 2:**
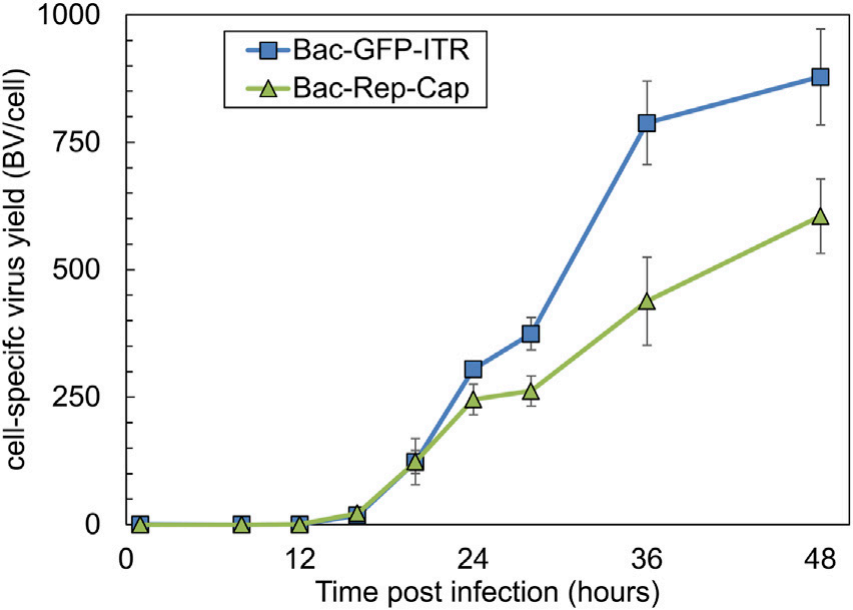
Baculovirus release kinetics after infection with an MOI of 10, visualized as BV released per cell (BV/cell) over time (0–48 hpi) for both Bac-GFP-ITR (blue squares) and Bac-Rep-Cap (green triangles). Data points are the mean value of three biological replicates ± Standard deviation.

The release kinetics of both Bac-GFP-ITR and Bac-Rep-Cap were assessed separately to determine how many baculovirus replication cycles are needed to reach complete co-infection for the asynchronous MOI experiments as well as potential baculovirus construct-related differences in replication. Sf9 cells were infected at a high MOI of 10, and after 1 h, the remaining BVs were removed by a complete medium refreshment. The dynamics of BV titers (BV/cell) were monitored over time on a per-cell basis (Figure 2). It was determined that the initiation of baculovirus budding into the supernatant occurred between 8 and 12 hpi. Differences in the maximum BV yield per cell were observed between the two baculovirus constructs. Specifically, Bac-GFP-ITR exhibited an average production of 880 BVs per cell, while Bac-Rep-Cap yielded a comparatively lower count of 605 BVs per cell at the 48 hpi time point.

### Effect of total MOI on rAAV production

To study the impact of total MOI on rAAV production, MOI values of 10:10, 0.1:0.1, and 0.001:0.001 (equal ratio) were implemented based on the measured BVs release curves.

Analyzing BV/cell values over time (Figure 2) reveals that an MOI of 0.1 for each baculovirus construct results in approximately 275 new BVs after 24 h. This means the MOI rises to about 30, and all cells become infected following the first replication cycle, even while the initially uninfected cells continue to proliferate. An MOI of 0.001 for each baculovirus construct means that, even after 48 h with 600–900 BVs released per cell, the MOI remains lower than one. Consequently, not all cells become infected after a single replication cycle, which suggests that two cycles are necessary to infect every cell.

**FIGURE 3:**
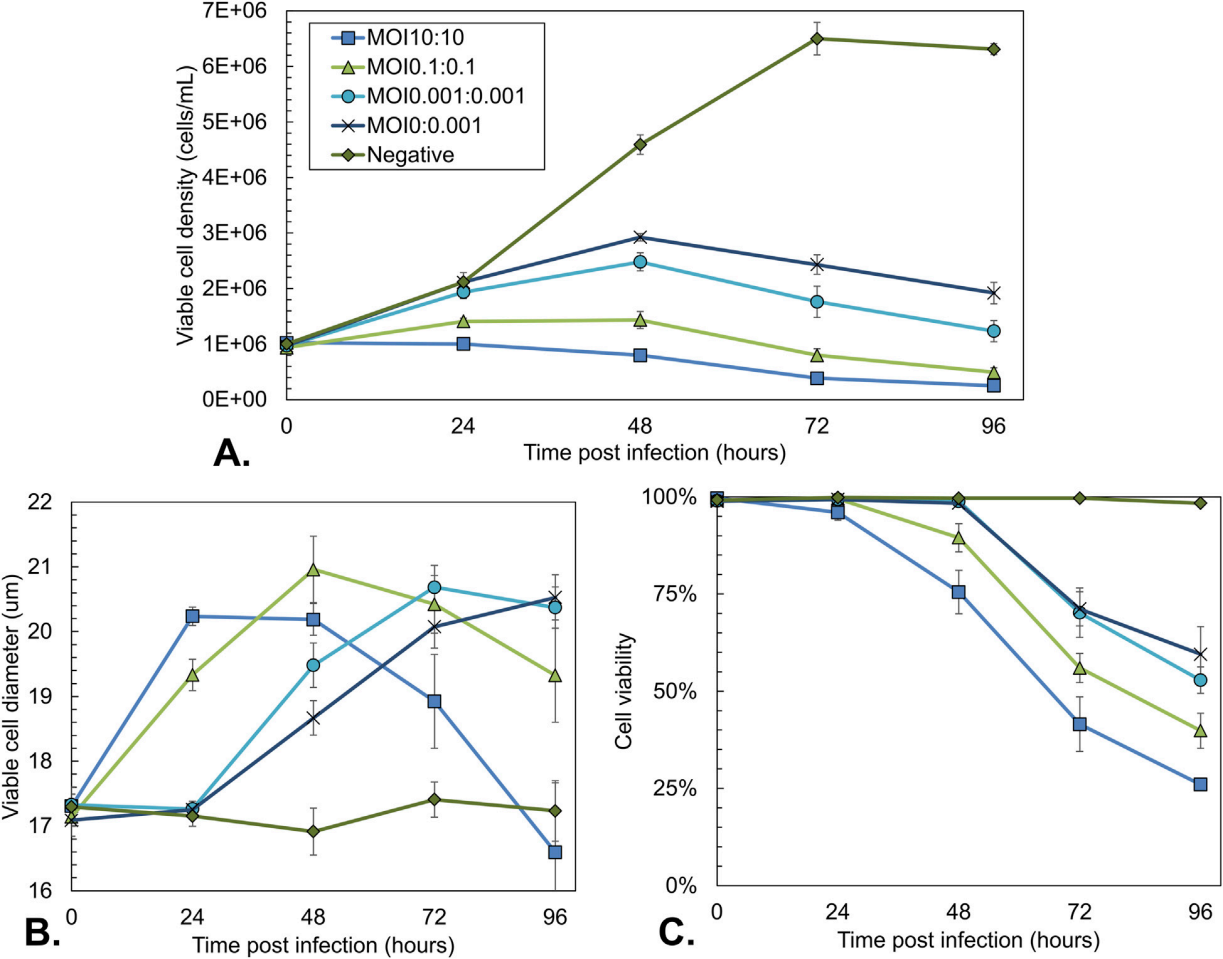
Sf9 cell culture characteristics during co-infection-based rAAV production using different total MOIs with an equal BV ratio. Uninfected Sf9 cells and a single infection with Bac-Rep-Cap (MOI 0.001) were used as a control. **(A)** Viable cell density over time. **(B)** Average diameter of viable cells over time. **(C)** Cell viability over time. Data points are the mean value of three biological replicates ± Standard deviation.

Viable cell density, average cell diameter, and viability were measured over time (Figure 3). Uninfected cells grew exponentially to the maximal density of 6–7 × 10^6^ cells/mL (Figure 3A) with a constant cell diameter of 17 µm (Figure 3B) and viability of 100% (Figure 3C). In line with expectations, all cells infected at a high MOI (10:10) became immediately infected, and no cell growth was observed, with the total cell density remaining constant at 1 × 10^6^ cells/mL while the cell viability declined over time (Figure 3C). At the same time, the cell diameter increased from 17 µm to over 20 µm. When infecting at lower MOI (0.1:0.1 and 0.001:0.001), the peak cell density increased because fewer cells became infected initially, and the uninfected cells had more time to grow. For MOI 0.1:0.1, a peak cell density of around 1.5 × 10^6^ cells/mL was reached. For 0.001:0.001, the cell density increased to 2.5 × 10^6^ cells/mL. In agreement with these observations, the decrease in viability and increase in cell diameter occurred later for the lower MOI values as multiple infection cycles were required (Figures 3B,C).

**FIGURE 4:**
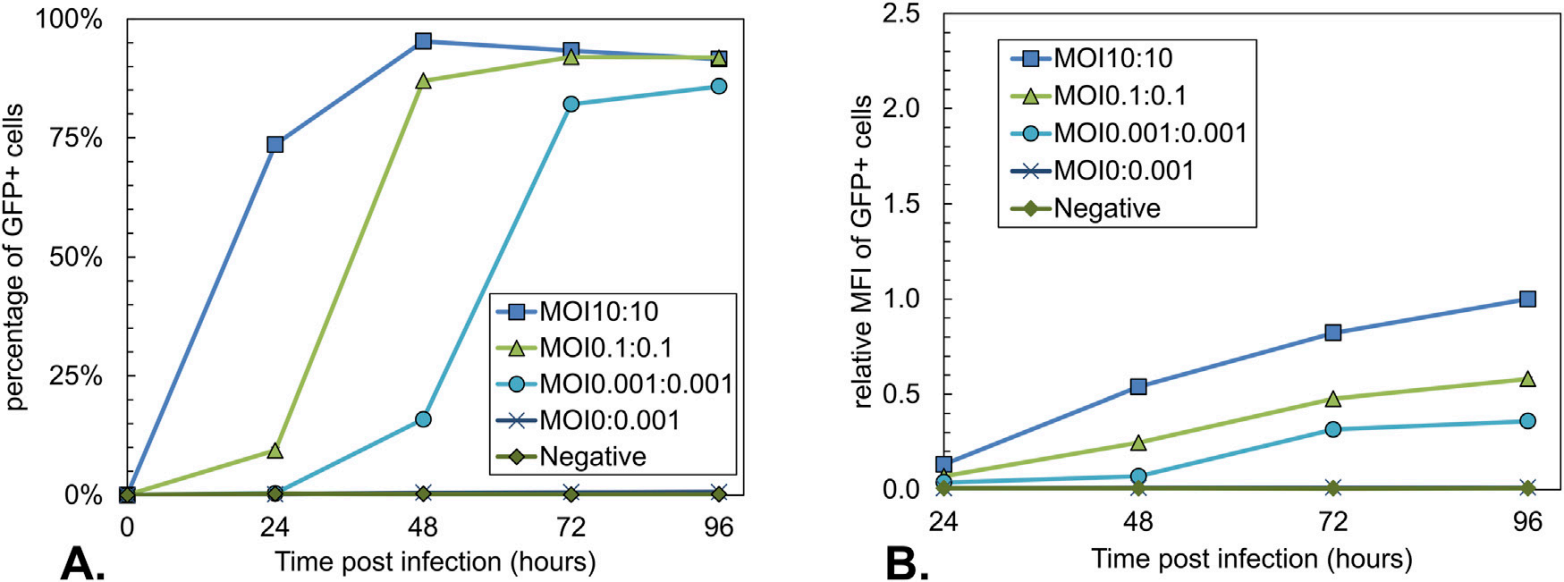
Analysis of the GFP expression during co-infection-based rAAV production using different total MOIs with an equal BV ratio. Uninfected Sf9 cells and a single infection with Bac-Rep-Cap (MOI 0.001) were used as a control. **(A)** Percentage of GFP-positive Sf9 cells over time. GFP-positive cells represent the Bac-GFP-ITR-infected cell population. **(B)** Relative Mean fluorescence intensity (MFI) of GFP-positive cells over time, normalized to the MOI10:10 condition at 96 hpi. Data points are the mean value of three biological replicates ± Standard deviation.

To follow the progression of the baculovirus infection, the percentage of GFP+ cells over time was analyzed for all conditions using flow cytometry (Figure 4A). This percentage is a measure of the cells infected with Bac-GFP-ITR. As expected, the Bac-Rep-Cap-only infected control condition is negative for GFP expression. In line with the anticipated additional replication cycles of the baculovirus infection, the time when most of the cells were positive for GFP was reached 24 h later when the total MOI was decreased by a factor of 100. Peak GFP+ cell percentage was lower for the MOI 0.001:0.001 condition at 96 hpi (86%, Figure 4A) compared to the higher MOI 10:10 and MOI 0.1:0.1 (95% and 92%, respectively; Figure 4A). The values for the relative MFI of the GFP+ cell population over time showed that decreasing the total MOI had a negative impact on the intensity of the GFP signal (Figure 4B), indicating a decrease in intracellular GFP content.

**FIGURE 5:**
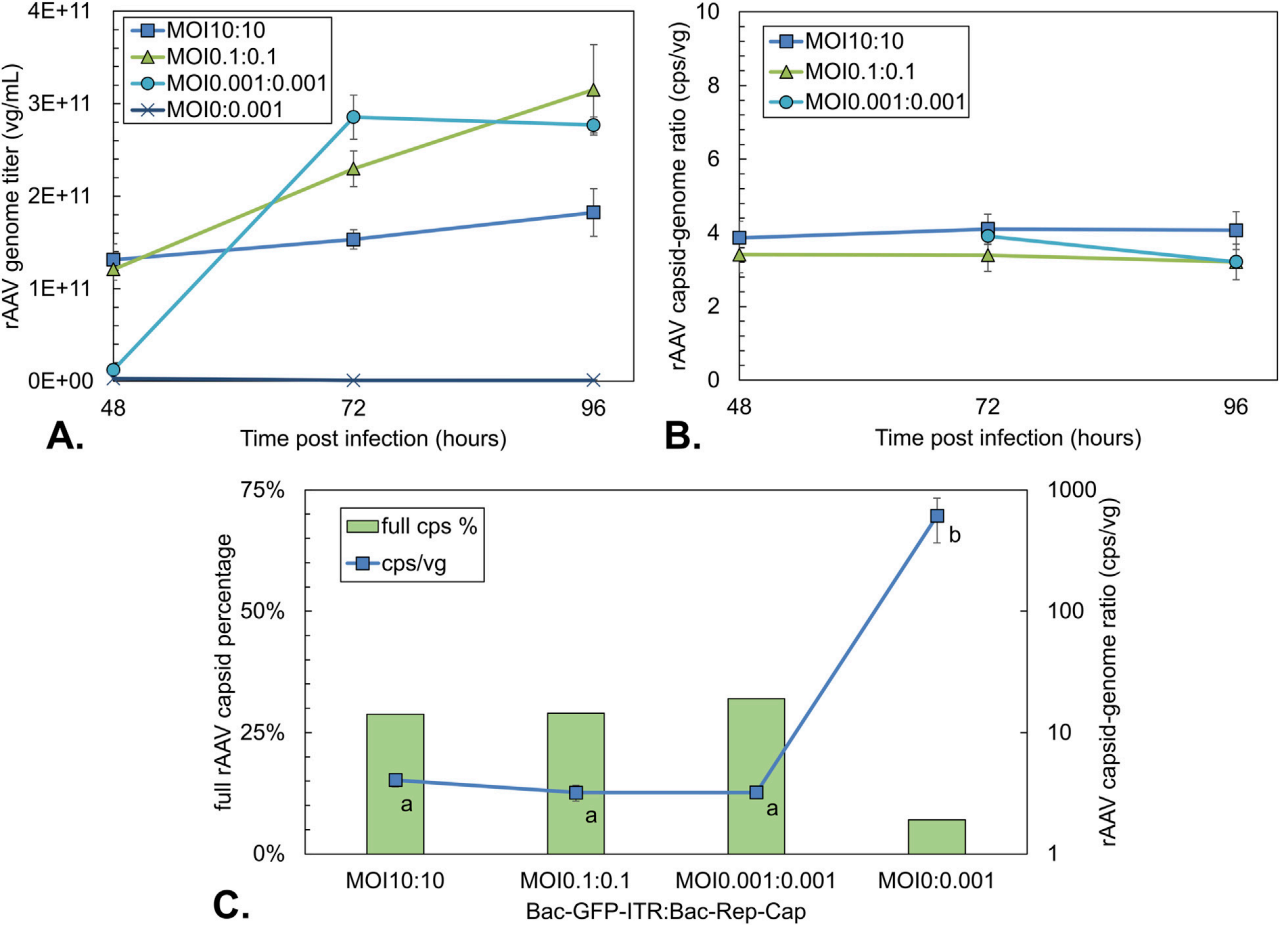
rAAV titers and rAAV quality during co-infection-based production using different total MOIs with an equal BV ratio. A single infection with Bac-Rep-Cap (MOI 0.001) was used as an empty rAAV capsid control. **(A)** rAAV genome titer over time based on qPCR titration. **(B)** rAAV capsid-to-genome ratio as determined by ELISA/qPCR over time. **(C)** Percentage of full rAAV capsids (green bars) and capsid-to-genome ratio (blue squares) at 96 hpi; determined by ELISA/qPCR or stunner data of pooled triplicates. Different letters indicate statistically significant differences between conditions (one-way ANOVA with Tukey’s *post hoc* test, p < 0.05). Data points are the mean value of three biological replicates ± Standard deviation.

Next, the effect of the total MOI on the volumetric yield of rAAV genomes was investigated (Figure 5A). At 48 h, as a result of synchronous infection, the production of rAAV genomes was highest post-infection for the MOI 10:10 condition, measuring 1.3 × 10^11^ vg/mL (Figure 5A). After 72 hpi and 96 hpi, the rAAV genome titer did not significantly differ for the highest MOI condition. In contrast, the MOI 0.1:0.1 condition followed a pattern of constant increase up to 96 hpi, reaching 3.1 × 10^11^ vg/mL. Because cell growth also continued, the final cell-specific yields were similar for the MOI 10:10 and 0.1:0.1 conditions (∼2.0 × 10^5^ vg/cell). For the lowest MOI condition, the genome titers were 10-fold lower at 48 hpi compared to other conditions, followed by an increase to 3 × 10^11^ vg/mL at 72 hpi, after which the titer stabilized. The final yield obtained per cell (1.2 × 10^5^ vg/cell) was around 40% lower than for the other conditions.

qPCR and capsid ELISA are commonly combined to estimate the proportion of full particles in rAAV preparations (Dorange and Le Bec, 2018; Kontogiannis et al., 2024). Considering both the rAAV genome as well as capsid yield over time, it can be observed that the capsid-to-genome ratio remains relatively constant around 3–4 cps/vg from 48 to 96 hpi for all conditions (Figure 5B). A constant percentage of around 30% full rAAV capsids at 96 hpi was found by Stunner measurements on purified (pooled triplicate) fractions of each total MOI condition (Figure 5C). Notably, the Bac-Rep-Cap-only condition (MOI 0:0.001) still yielded 7% full particles as determined by the stunner despite the absence of a rAAV genome. The capsid-to-genome ratio for the Bac-Rep-Cap-only condition was determined to be around 600 cps/vg, roughly two orders of magnitude higher than co-infection conditions.

### Impact of BV ratios in synchronous infection on rAAV production

**FIGURE 6:**
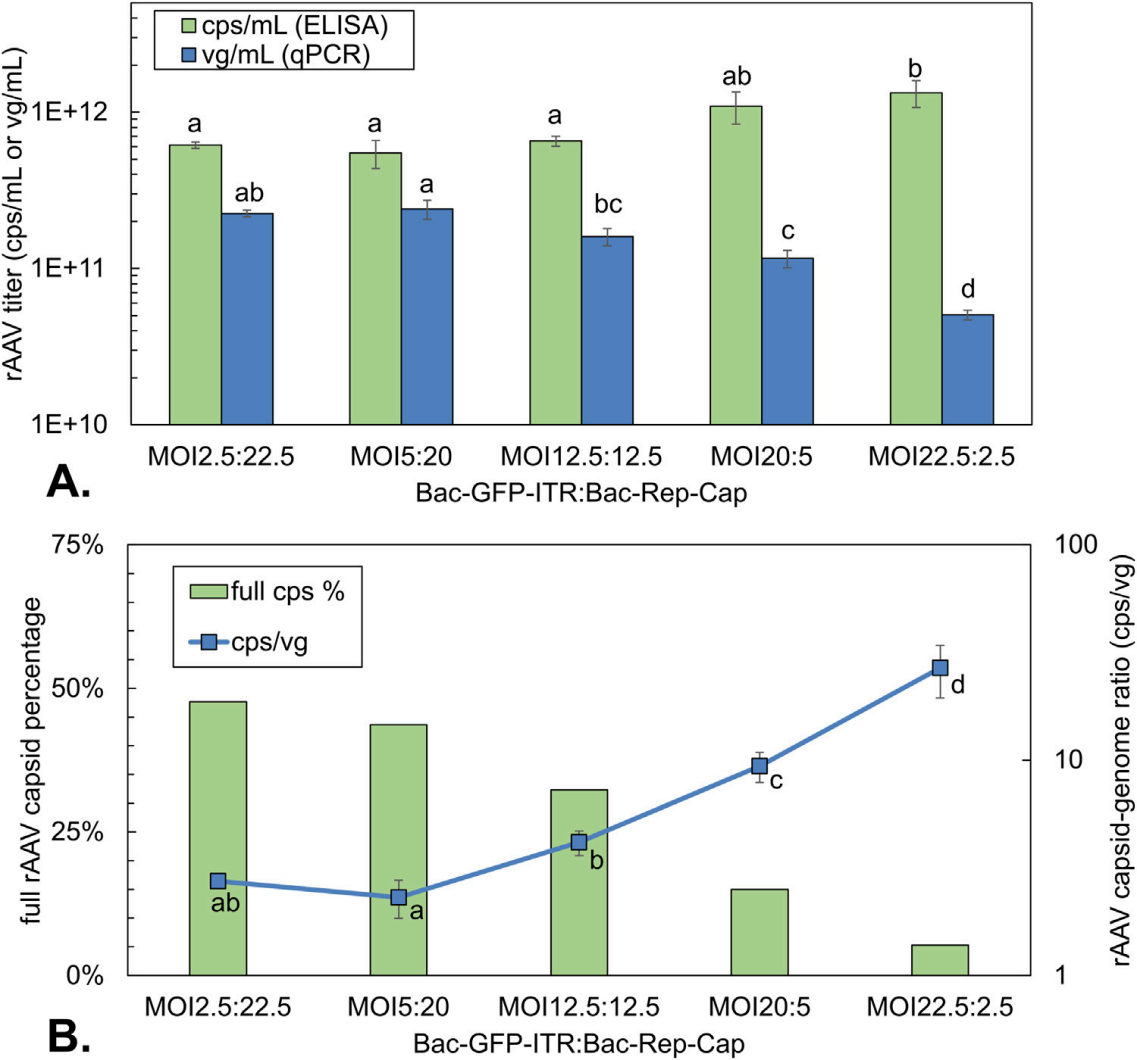
rAAV titers and rAAV quality of co-infection-based production using a constant total MOI of 25 with different BV ratios. **(A)** Volumetric rAAV capsid (green bars) and genome titer (blue bars) at 72 hpi. **(B)** Percentage of full rAAV capsids (green bars) determined on pooled triplicates of 72 hpi by Stunner and capsid-to-genome ratio (blue squares) determined by ELISA/qPCR at 72 hpi. Different letters indicate statistically significant differences between conditions within the same analytical method (one-way ANOVA with Tukey’s *post hoc* test, p < 0.05). Bars and data points are the mean value of three biological replicates ± Standard deviation.

Building on the evaluation of the total MOI used for rAAV production, while maintaining an equal ratio, the impact of varying the BV ratio under synchronous high MOI infection conditions was studied (Figure 6). All conditions were infected at a viable cell density of 1 × 10^6^ cells/mL with a similar total MOI of 25 (actual MOIs of 2.5:22.5, 5:20, 12.5:12.5, 20:5, 22.5:2.5; Bac-GFP-ITR: Bac-Rep-Cap).

When Bac-Rep-Cap was equal to or higher than Bac-GFP-ITR, AAV capsid yields reached around 6 × 10^11^ cps/mL at 72 hpi (Figure 6A). However, when Bac-GFP-ITR was the dominant virus in the co-infection, the capsid yield per cell doubled to around 1.2 × 10^12^ cps/mL. The yield of encapsidated rAAV genomes increased with the amount of Bac-Rep-Cap, up to 2.5 × 10^11^ vg/mL for an MOI of 5:20. However, increasing the amount of Bac-Rep-Cap (up to a ratio of 2.5:22.5) did not lead to any further enhancement in the production of the encapsidated genome. Regarding the capsid-to-genome ratio, a 10-fold increase was observed when comparing the condition with the largest fraction of Bac-Rep-Cap (MOI 2.5:22.5; 3 cps/vg) to the smallest fraction (MOI 22.5:2.5; 30 cps/vg) (Figure 6B). The trend of the capsid-to-genome ratio was confirmed with Stunner measurements on purified rAAV samples also showing a 10-fold decrease of the percentage of full rAAV capsids (50%–5%, Figure 6B). Notably, for the 1:1 ratio (12.5:12.5 of Bac-GFP-ITR: Bac-Rep-Cap), a similar full rAAV capsid yield was found compared to results obtained when varying the total MOI, maintaining a 1:1 ratio (Figure 5C: 29%–32%; Figure 6B: 32%).

**FIGURE 7:**
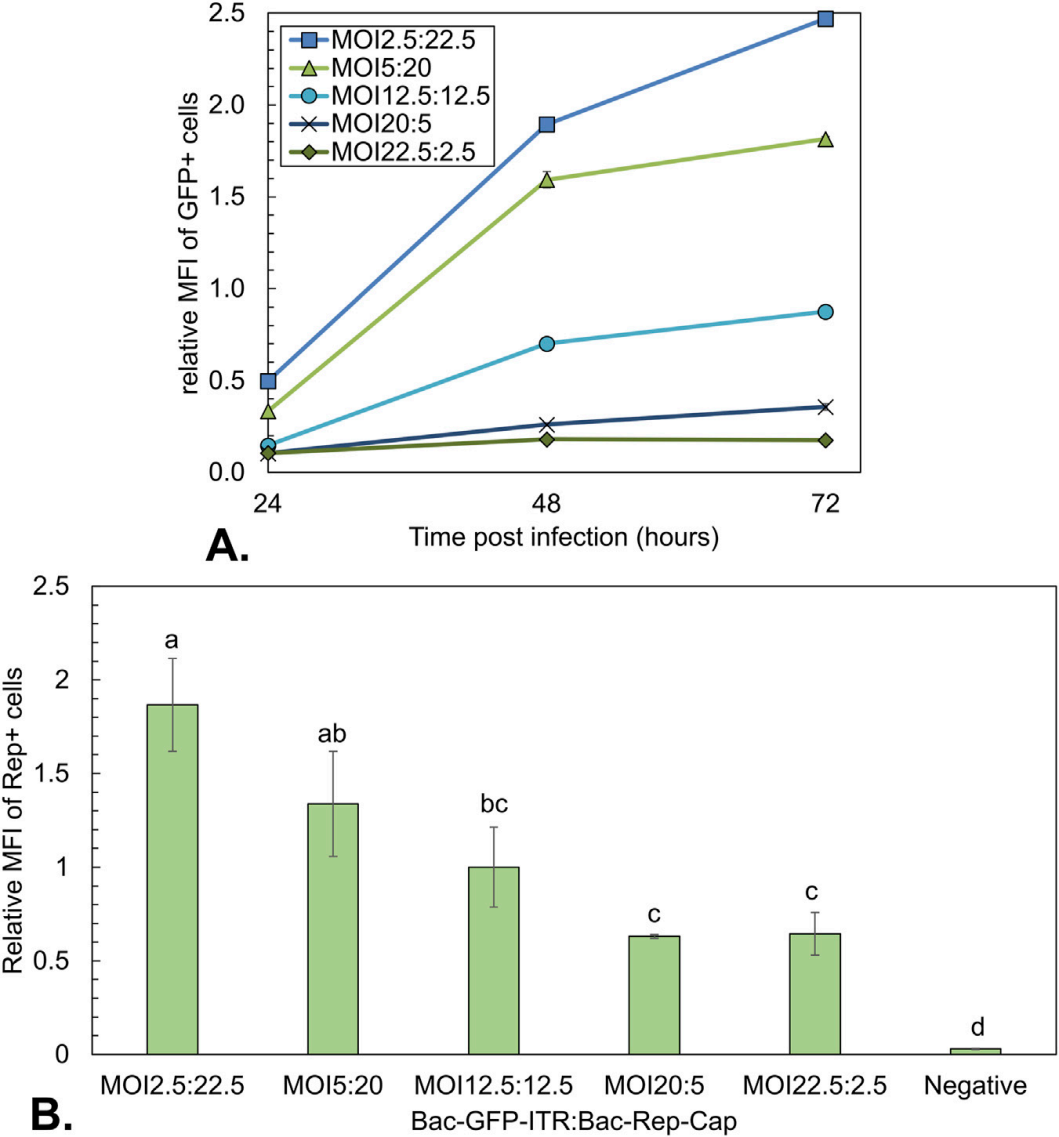
Analysis of the GFP expression during co-infection-based rAAV production using a constant total MOI of 25 with different BV ratios. **(A)** Mean fluorescence intensity (MFI) over time of GFP-positive cells. Data was normalized to the MOI10:10 condition at 96 hpi (Figure 4). **(B)** MFI at 48 hpi of Rep positive cells (gated in 780 nm bandpass filter). Different letters indicate statistically significant differences between conditions (one-way ANOVA with Tukey’s *post hoc* test, p < 0.05). Bars and data points are the mean value of three biological replicates ± Standard deviation.

Apart from the rAAV yield, the infected Sf9 cells were analyzed by flow cytometry on the expression levels of GFP and Rep proteins. GFP was measured directly, while the intracellular Rep proteins were stained with an antibody-fluorochrome conjugate in fixed and permeabilized Sf9 cells (Figure 7). The uninfected control showed no positive signal for Rep staining or fluorescence for GFP. Based on the relative MFI of viable cells in the 525 nm bandpass filter, relative GFP expression levels were inferred (Figure 7A). The MFI was plotted relative to the final MFI of the MOI 12.5:12.5 condition. It was observed that the GFP expression increases for all infected conditions over time. The biggest increase in MFI was between 24 and 48 hpi for all infected samples. Furthermore, the GFP expression level increased with an increasing fraction of Bac-Rep-Cap. The highest MFI value was observed for the co-infection conditions where the Bac-Rep-Cap baculovirus is most prominently present (MFI around 2.5-fold higher at 72 hpi compared to the 1:1 ratio of BV). The relative MFI for fixated Rep-stained cells at 48 hpi was normalized to the MFI values of the MOI 12.5:12.5 condition. There was a clear rise in the relative expression of total Rep proteins with an increase of Bac-Rep-Cap in the co-infection. Thus, increased Bac-Rep-Cap input is linked to higher Rep and GFP expression, which correlates with the observed increase in AAV genome production.

### Impact of BV ratios on rAAV production under asynchronous MOI conditions

To assess the impact of an additional baculoviral replication cycle on the effects of the BV ratio in rAAV production, the previous experiment at high MOI was repeated using the same culture conditions, but now with a total MOI of 0.2 (actual MOIs of 0.02:0.18, 0.04:0.16, 0.1:0.1, 0.16:0.04, 0.18:0.02). As shown in the total MOI experiment (Figures 3–5), this low MOI resulted in an additional replication cycle and delayed production compared to synchronous infection. Based on the results presented in Figure 5, rAAV yields were compared at 96 hpi for this experiment. All conditions reached a maximum cell density of 1.5 × 10^6^ cells/mL before cell division was halted due to infection.

**FIGURE 8:**
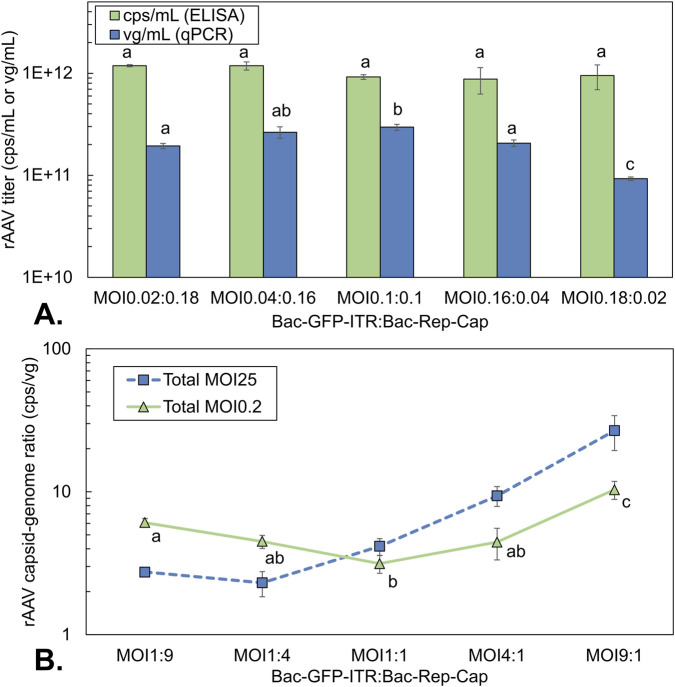
rAAV titers and rAAV quality of co-infection-based production using a constant total MOI of 0.2 with different BV ratios. **(A)** volumetric rAAV capsid (green bar) and genome yield (blue squares) at 72 hpi. **(B)** capsid-to-genome ratio at a total asynchronous MOI of 0.2 (green triangles) compared to the capsid-to-genome ratio at a total MOI of 25 (orange squares, also Figure 6B) using different ratios of baculovirus; determined by ELISA/qPCR. Different letters indicate statistically significant differences between conditions (one-way ANOVA with Tukey’s *post hoc* test, p < 0.05). Bars and data points are the mean value of three biological replicates ± Standard deviation.

The volumetric yield of rAAV capsids and genomes was determined with different BV ratios at 96 hpi using an asynchronous infection (MOI 0.2) (Figure 8). Capsid yield was comparable for all conditions, with values around 1 × 10^12^ cps/mL (Figure 8A). There seemed to be a slightly higher capsid yield for the conditions where Bac-Rep-Cap predominates co-infection (∼30%, not statistically significant). For the rAAV genome yield, the highest value is obtained with a 1:1 ratio, while an excess of one of the two viruses decreases the genome yield. This contrasts with the observations at synchronous infection conditions, where an excess of Bac-Rep-Cap increases rAAV genome titers (Figure 6A). Likewise, the capsid-to-genome ratio also increased when the baculovirus constructs are added in unequal amounts (from around 3 cps/vg to up to 10 cps/vg, Figure 8B).

**FIGURE 9:**
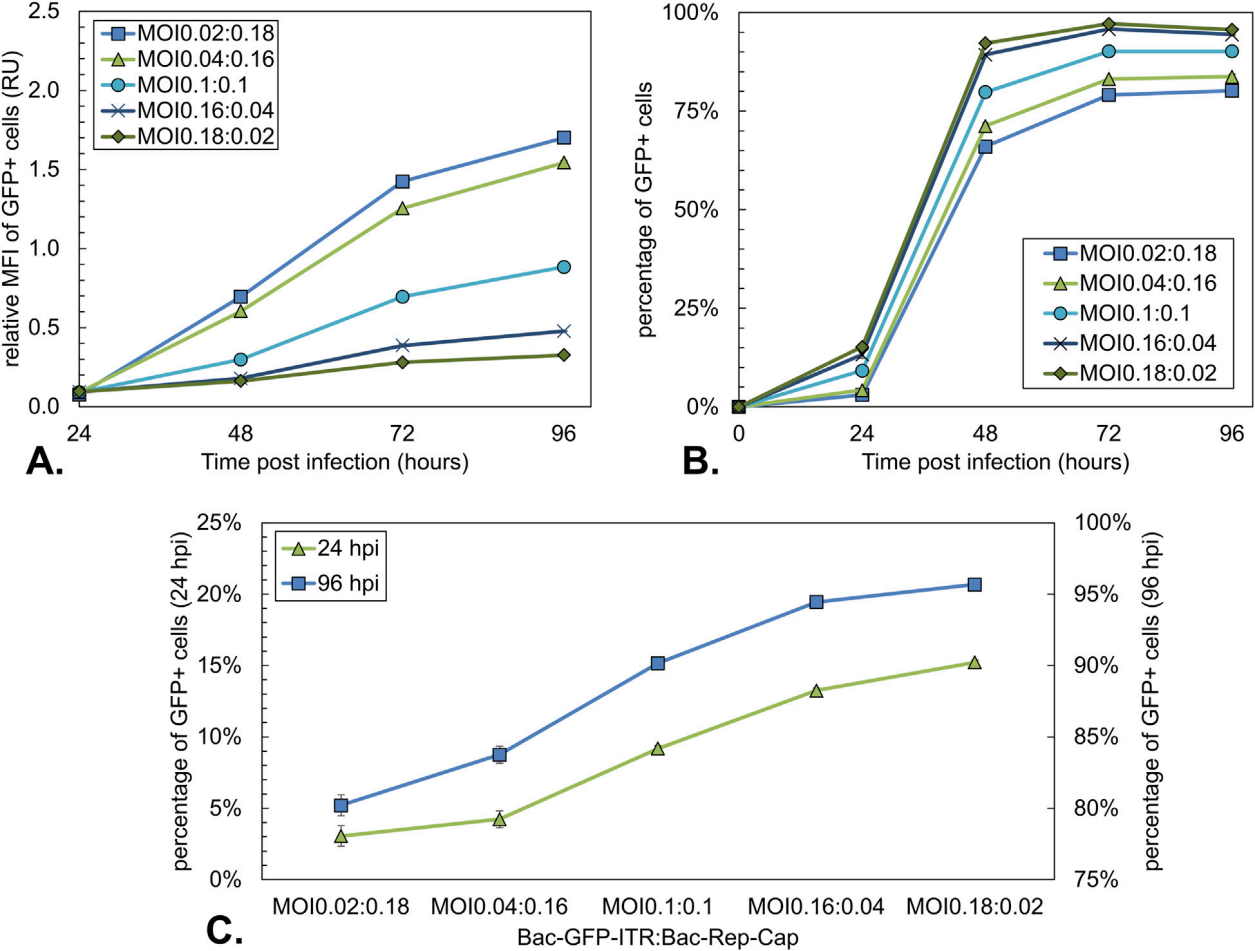
Analysis of the GFP expression during co-infection-based rAAV production using a constant total MOI of 0.2 with different BV ratios. **(A)** Mean fluorescence intensity (MFI) over time of GFP-positive cells. Data were normalized to the MOI10:10 condition at 96 hpi (Figure 4). **(B)** Percentage of GFP-positive Sf9 cells over time, where GFP-positive cells represent the Bac-GFP-ITR-infected cell population. **(C)** Percentage of GFP-positive Sf9 cells at 24 hpi (green triangles) and 96 hpi (blue squares). Bars and data points are the mean value of three biological replicates ± Standard deviation.

GFP expression levels were again assessed based on relative MFI. The MFI of the GFP-positive cells (Figure 9A) showed an increasing MFI with the amount of Bac-Rep-Cap, as also seen for the synchronous infection (Figure 7A). The 1:1 ratio of both the synchronous and asynchronous infection showed a similar maximum in MFI (72 hpi in Figure 7A, and 96 hpi in Figure 9A). However, the conditions with an excess of Bac-Rep-Cap had a lower relative MFI, and conditions with an excess of Bac-GFP-ITR had a higher relative MFI compared to the synchronous infection conditions (Figure 7A).

Since not all cells in the asynchronous infected conditions were directly infected, the percentage of GFP+ cells over time was evaluated. The Percentage of GFP+ cells increased from 24 hpi onwards and reached a plateau between 48 and 72 hpi (Figure 9B). At 24 and 96 hpi, GFP+ cell percentages ranged from 3% to 15% and 80%–96%, respectively, with the percentage of GFP+ cells decreasing with an increasing ratio of Bac-Rep-Cap used for infection (Figure 9C).

## Discussion

Low MOI conditions need additional baculovirus replication cycles to completely infect the insect cell culture during the rAAV production process. The determination of an optimal MOI is ideally guided by the BV release kinetics of the specific baculovirus construct under defined conditions. Given that two distinct baculovirus constructs are needed for generating rAAVs and recognizing potential variations in BV production per infected cell, the release kinetics of both Bac-GFP-ITR and Bac-Rep-Cap constructs were assessed. The cell-specific BV yield reported in literature is highly variable, ranging from 3 to 400 BV/cell, depending on the medium, cultivation vessel, and cell line used (Carinhas et al., 2010; Guardalini et al., 2022; Wickham et al., 1992). We observe yields of more than 600 BV/cell. It should be considered that the conditions used for obtaining the release kinetics were non-limiting by employing a relatively low CCI of 1 × 10^6^ cells/mL. Potentially, the nutrient-rich, chemically defined medium used here allows for increased BV production compared to media systems used in literature. Additionally, different BV titration assays are mentioned in literature. Here, we determine titers based on a flow cytometric assay detecting the expression of the BV GP64 protein. Differences between the classical endpoint dilution assays and the method implemented here cannot be ruled out (Lothert et al., 2023).

Except for a mutation in the very late P10 gene, the baculovirus backbones used for rAAV production are similar in sequence. Therefore, differences in the BV release kinetics observed are likely the result of the expression of the rAAV transgenes. A 30% reduction in maximum BV/cell for Bac-Rep-Cap was measured compared to Bac-GFP-ITR. In addition to that, a more rapid decline in viability for Bac-Rep-Cap infected cells compared to Bac-GFP-ITR was observed (Cell viability at 48 hpi; Bac-Rep-Cap: 37%, Bac-GFP-ITR: 67%). The toxicity of the Rep proteins, especially Rep78, has been acknowledged, and this is in line with the differences in release kinetics and viability profile obtained with Bac-Rep-Cap infection (Moreno et al., 2022; Urabe et al., 2006).

The impact of total MOI on rAAV production with equal amounts of both baculovirus constructs was evaluated by incorporating one or two additional baculovirus replication cycles in the production culture. One of the hypothesized problems of low MOI co-infection, due to the asynchronous nature, is the occurrence of superinfection exclusion, a phenomenon where infected cells cannot be infected with a secondary virus after the primary infection is fully established. The effect has been described for baculoviruses in literature and occurs before new BV are budding from the cell (Beperet et al., 2014). This would mean that in asynchronous infection strategies, cells initially infected by one baculovirus construct cannot properly become co-infected and would, therefore, be unable to produce rAAV particles containing a genome.

With one additional baculovirus replication cycle (MOI 0.1:0.1), higher volumetric titers, but comparable final cell-specific yields of rAAVs (2 × 10^5^ vg/cell), were obtained as with a synchronous infection. The results suggest that low MOI conditions for rAAV production yield cell-specific productivities similar to those achieved with high MOI conditions. This aligns with Mena et al. (2010), who observed comparable outcomes with a Triple-Bac system at both low and high MOI using equal baculovirus ratios.

Regarding superinfection exclusion, only a minor loss of ∼10% of cells able to produce rAAV capsids is expected in the MOI 0.1:0.1 condition, which falls within the measurement accuracy of the rAAV titrations. However, the maximum percentage of GFP+ cells is slightly lower for the MOI 0.1:0.1 condition than the MOI 10:10 condition (92%–95%, respectively; Figure 4A).

The volumetric yield of rAAV was comparable for asynchronous infections with one and two replication cycles (MOI 0.1:0.1 and MOI 0.001:0.001). However, the variation in maximum cell densities indicates a reduced cell-specific yield at the lower MOI condition. When looking at the GFP+ cell percentages over time, we also observe a reduction in the maximum GFP+ cell percentage for MOI 0.001:0.001 compared to MOI 10:10 (86%–95%).

For the MOI 0.001:0.001, the decrease in yield due to superinfection is hard to predict since it depends on the BV release kinetics and MOIs reached after the first replication cycle. Although the reduction of GFP+ cells indicates superinfection exclusion to some extent, it alone cannot explain the drop in cell-specific rAAV yield. It is more likely that the drop in cell-specific yield is caused by the continued cell growth to 2.5 × 10^6^ cell/mL and the limited capacity of the media system used here, for several reasons: First of all, the single infected Bac-Rep-Cap control (MOI0:0.001) has a lower AAV capsid yield in combination with a higher final cell density indicating the limited medium capacity as the primary cause as there is no co-infection (Figure 3A). Secondly, experiments to determine the cell density at which cell-specific yield drops using the medium in this study show the maximum capacity is reached between 2–4 × 10^6^ cells/mL. To clearly separate the effects of nutrient limitation from those of MOI, future experiments could use a fed-batch or dilution approach for low MOI infections, thereby reducing the impact of post-infection cell growth. When the cell density is kept below a threshold, low MOI infections demonstrate efficient rAAV production with less virus inoculum. These strategies are ideal for large-scale manufacturing because they lower seed requirements and simplify upstream process logistics.

One aspect not considered in this study is the packaging of DNA into the AAV capsids, which is not part of the rAAV genome. The ITR sequence-specific qPCR analysis reveals a 100-fold lower signal for AAV genome content in Bac-Rep-Cap-only infected cells compared to co-infected conditions, as there is no ITR sequence present. However, the Stunner reading, which is not dependent on a specific DNA sequence, estimates that 7% of the particles contain DNA. Complementary analyses with ITR-specific qPCR and AAV capsid ELISA confirm that DNA-containing particles in co-infected setups are properly packaged rAAV vectors. Consequently, the signal seen with Stunner in the Bac-Rep-Cap-only control probably indicates non-specific encapsidation and does not affect the interpretation of full capsid ratios across different conditions. Non-specific DNA packaging by Rep proteins has been previously observed for Rep-Cap sequences in the absence of ITRs (Nony et al., 2003). While Kondratov et al. (2017) reported <1% host or baculoviral DNA in capsids under standard co-infection and Penaud-Budloo et al. (2017) found less than 2% of baculoviral DNA contaminants, they did not investigate ITR-deficient conditions. The detected signal might also originate partly from non-packaged residual Sf9 or baculoviral DNA that was not eliminated during purification, which could contribute to the DNA content observed by the Stunner. Future research employing techniques such as analytical ultracentrifugation (AUC) or sequencing could clarify the composition and origin of the packaged DNA, thereby enhancing our understanding of how non-template DNA is packaged by Rep in the BEVS when no packaging signals (ITRs) are available.

Apart from the total MOI, the effect of the ratios between the two baculovirus constructs was investigated in this study. The total MOI was kept constant in the experiments to exclude effects of overall infection pressure on rAAV yield. To assume synchronous infection, a total MOI of 25 for co-infection was implemented, which means that the lowest individual MOI of 2.5 yields an infection percentage of 90.2% according to the Poisson distribution. In other words, even at the MOI ratios 2.5:22.5 and 22.5:2.5, most cells will be co-infected by the two different baculovirus constructs.

The production of encapsidated rAAV genomes decreases with a decreasing amount of Bac-Rep-Cap, which is expected since genome replication and packaging occur by the Rep78 and Rep52 proteins expressed by this construct. GFP on Bac-GFP-ITR is constitutively expressed in Sf9 cells by a non-baculoviral promotor (CMV) at a low level. However, GFP expression appears to correlate with rAAV genome replication. When co-infected, the relative MFI of the GFP expression increases with an increased fraction of Bac-Rep-Cap in the co-infection and with the encapsidated rAAV genome titer.

As mentioned, by increasing the Bac-Rep-Cap fraction in synchronous co-infection with Bac-GFP-ITR, we observe an increase in encapsidated rAAV genomes. This can be explained by the higher expression levels of the Rep proteins, as confirmed by the intracellular antibody staining on total Rep proteins (Figure 7B), which are responsible for rAAV genome production and packaging. Interestingly, at higher Bac-Rep-Cap fractions, the rAAV capsid yield is lower compared to when Bac-GFP-ITR is supplied in excess. The capsid proteins, VP1, VP2, and VP3, are on the same construct as the Rep proteins so one would expect an increase in capsids, just like observed for Rep. A possible explanation for the decrease in cell-specific capsid yield with the increasing amount of Bac-Rep-Cap could be transgene toxicity, which limits the production of VPs or their assembly into capsids. Multiple literature reports on mammalian production systems indicate the adverse effects of high Rep78 expression on the production of rAAVs (Grimm et al., 1998; Li et al., 1997). Also, Rep78 expression may indirectly interfere by amplifying the rAAV genome and consequently increasing the GFP expression. This increase in GFP/GOI expression might, in turn, compete with VP and Rep for cellular protein synthesis machinery, available energy, or amino acids (Destro et al., 2023). Mechanistic modeling of the rAAV production process with the Dual-bac system by Destro et al. (2023) indicates that transgene expression from the free rAAV genome is expected to inhibit total rAAV production (both empty and full capsids). Based on the results obtained by studying rAAV production in a synchronous co-infection, the highest percentage of full rAAV capsids (up to 50%) is obtained when supplying an excess Bac-Rep-Cap (2.5:22.5).

Varying the ratio of BV in asynchronous, low MOI co-infections results in a different outcome for rAAV yield compared to high MOI conditions. To follow infected cell populations over time, we evaluated GFP expression from the Bac-GFP-ITR construct. Increasing the Bac-GFP-ITR MOI leads to a higher percentage of GFP+ cells during the initial infection at 24 hpi. When the percentage of GFP+ cells reaches a plateau between 48 and 72 hpi, the proportion of non-fluorescent cells correlates with the initial Bac-Rep-Cap MOI.

These observations are consistent with the occurrence of superinfection exclusion, meaning that cells that initially were infected with Bac-Rep-Cap do not become infected by the Bac-GFP-ITR during the following infection cycle, or at least this does not lead to GFP expression.

Regarding the release curves of both baculovirus constructs up to 24 hpi, the amount of BVs released per cell is relatively similar, approximately 275 particles per cell. This would mean that the minimal individual MOI is 5.5 BV per cell after 24 hpi, and full co-infection of the remaining cells is expected. It should be noted that BV release kinetics were evaluated using an MOI of 10; however, we did not assess if the BV release from a cell is affected by viral load.

The lowest capsid-to-genome ratio in asynchronous infection is when both Bac-GFP-ITR and Bac-Rep-Cap are added in equal amounts. The capsid-to-genome ratio of around 3 cps/vg agrees with the results of the earlier experiment with equal ratios at different total MOIs. We observe an increase in capsid titer (∼30%) at conditions with a higher fraction of Bac-Rep-Cap. The rAAV genome titer in these conditions does not increase correspondingly but shows a declining trend. An excess of the Bac-Rep-Cap virus at low MOI conditions does not increase the full capsid percentage as seen with synchronous infection conditions. This could be explained by superinfection exclusion, where a lower initial fraction of Bac-GFP-ITR is present at higher Bac-Rep-Cap fractions. This results in a percentage of cells only infected with Bac-Rep-Cap during the initial infection (∼16%–18% of the cells), and these cells would only be able to produce empty capsids without subsequent co-infection due to superinfection exclusion.

With synchronous infection, an optimal ratio of 1:9 for Bac-GFP-ITR to Bac-Rep-Cap was found, resulting in up to a 50% full capsid fraction, which is higher than the maximum value obtained at asynchronous infection. The ‘tweaking’ of rAAV-related protein expression levels based on BV ratios at synchronous MOI might prove challenging to implement in a low MOI strategy. That does not mean low MOI cannot be used to produce rAAVs using the Dual-Bac rAAV production platform. A low MOI strategy reduces the virus stock to be added to the production volume by more than a factor of 100, and depending on the combination of MOI and CCI, similar cell-specific rAAV yields can be obtained as with high MOI, though product quality may differ based on the ratio of the two baculovirus constructs.

Superinfection exclusion may impact yield differently depending on the exact asynchronous MOI implemented. At a total MOI of 0.2, approximately 20% of insect cells are infected. However, most of the infected cells receive only a single baculovirus construct and may not be reinfected during the release of secondary BVs. In contrast, with a lower MOI of 0.02, just 2% of cells become infected, which may increase the co-infection percentage in the subsequent infection cycle and improve yield. Supporting this notion, the findings of Negrete et al. (2007) show a decrease in AAV yield at an MOI of about 1, while higher titers were achieved at both lower and higher MOIs.

It should be noted that the experiments in our study were mostly performed at CCIs below the maximum capacity of the medium for experimental purposes, such as comparing different total MOIs. This means that the volumetric yields may be increased by optimizing CCIs for specific MOI conditions.

Co-infection, and even more so with the incorporation of additional replication cycles, requires a pure stock of the BVs. The presence of BVs that do not contain all transgenes can interfere significantly with product formation. Aucoin et al. (2006) and Kohlbrenner et al. (2005) show the negative effect of these BVs on Rep expression as well as rAAV production and refer to it as the passaging effect. The presence of these ‘empty’ BVs will result in more complex infection conditions. It may explain literature reports indicating lower cell-specific yields at asynchronous infection that cannot be explained by nutrient limitation. For these reasons, the BV stocks used in this study were plaque-purified, the insert length was verified by end-point PCR, and VP, Rep, and GFP expression was checked by Western blotting and fluorescence microscopy.

Optimization of rAAV production lies partly in the strategy of baculovirus infection and partly in balancing the expression levels of AAV-related proteins. To avoid the potential effects of superinfection exclusion and BV ratio, ideally, a stable Single-Bac system would be employed where the expression of Rep52, Rep78, and VPs are tuned appropriately both in intensity and time-wise, by evaluating different promoter sequences, to reach maximal cell-specific yield under non-limiting medium conditions. While the Single-Bac system is ideal, the Dual-Bac platform remains important for industrial rAAV production. Since Rep and Cap are co-expressed from a single baculovirus, their levels cannot be adjusted independently like in the Triple-Bac system through MOI. Therefore, even in the Dual-Bac system, promoter engineering would be an effective way to fine-tune AAV protein expression.

## Conclusion

Our research on the effect of the BV ratio and total MOI in the Dual-Bac AAV production system has shed light on crucial factors influencing rAAV yield and quality. The highest full rAAV capsid percentage can be obtained by using a synchronous co-infection strategy where Bac-Rep-Cap is supplied in excess compared to Bac-GFP-ITR (at least 4:1, respectively). Increasing the ratio of Bac-Rep-Cap to Bac-GFP-ITR enhances the expression levels of the Rep proteins involved in rAAV genome replication and packaging, thereby increasing the number of filled rAAV capsids. Asynchronous infection strategies (low MOI) can be implemented, where the optimum ratio is when both baculovirus constructs are added in equal amounts. Although the maximum fraction of full rAAV particles was lower than in synchronous infection (approximately 30% compared to 50%), asynchronous infection offers advantages in terms of virus inoculum size and process scalability. The difference between synchronous and asynchronous infections is thought to result from the effect of the BV release kinetics and potentially superinfection exclusion hampering secondary infections. When maintaining a ratio of one for both BVs, lowering the total MOI has little effect on the quality of rAAVs produced (capsid-to-genome ratio). In contrast, at low MOI conditions, higher volumetric yields were obtained as a consequence of continued cell growth, resulting in higher peak cell concentrations. Beyond a specific peak cell density, the cell-specific rAAV yield declines, likely because of the nutrient capacity of the culture medium.

## Data Availability

The raw data supporting the conclusions of this article will be made available by the authors, without undue reservation.

## Abbreviations

AAV, Adeno-associated virus; AcMNPV, Autographa californica multiple nucleopolyhedrovirus; BEVS, baculovirus expression vector system; BV, budded virus; Cap, capsid proteins (Viral protein 1, 2, and 3); CCI, cell concentration at infection; cps, rAAV capsids; GFP, green fluorescent protein; GOI, gene of interest; MFI, mean fluorescence intensity; MOI, multiplicity of infection; Rep, Replicase protein (52 and/or 78); rAAV, recombinant AAV vector; Sf9, *Spodoptera frugiperda* cell line *9*; vg, rAAV genomes; VP, viral capsid proteins.
